# Supplementary material for: Quantum billiards with correlated electrons confined in triangular transition metal dichalcogenide monolayer nanostructures
Source: Nat Commun. 2021 Jun 18;12:3793. doi: 10.1038/s41467-021-24073-0 (PMC8213767; doi:10.1038/s41467-021-24073-0)
Supplement: Supplementary file 1 — Supplementary information [file 41467_2021_24073_MOESM1_ESM.pdf]

Supplementary Information

Quantum billiards with correlated electrons confined in triangular transition metal dichalcogenide monolayer nanostructures

Jan Ravnik, Yevhenii Vaskivskyi, Jaka Vodeb, Polona Aupič, Igor Vaskivskyi, Denis Golež, Yaroslav Gerasimenko, Viktor Kabanov, Dragan Mihailovic

Supplementary note 1. Experimental details

The transformation was performed directly within the UHV chamber of an Omicron Nanoprobe LT STM as shown schematically in Supplementary Figure 1. We use an amplified 800 nm Ti:sapphire laser at a repetition rate of 100kHz to induce the single-layer transformation in the sample. The Gaussian laser pulses have a duration of 40 fs and peak fluence of up to 12 mJ/cm2. An optical microscope and a vertical SEM microscope are used for alignment and wide area search on the sample respectively.

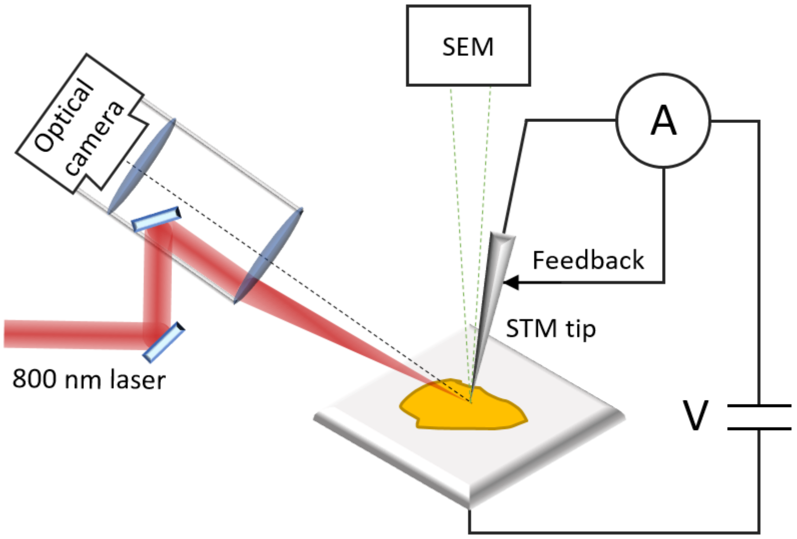

Supplementary Figure 1. Schematic representation of the experimental apparatus. Above the scanning tunneling microscope (STM) we have a scanning electron microscope (SEM) which enables us precise tip positioning on the sample. At the side of the STM there is a quartz window, through which we introduce the laser pulses. In case of imaging, we use the constant current mode, with the feedback controlling the height of the tip. For the I-V curves, the feedback is turned off.

Unlike with UHV in-situ thermal conversion[1], the laser transformation process results in triangular domain formation required for this study. The properties of single polytype transformed layers of 1H-TaS_2 on 1T-TaS_2 were reported previously[2]. The conditions for creating triangles are found to be very delicate. The transformed areas vary between the experiments. In some cases, we observe the transformation of the full layer, leading to the temperature-dependent interference patterns between the top two layers[2]. In other cases, the surface is only partially transformed, with the ratio of the transformed area with respect to the observed area of the sample ranging anywhere between 0 and 1. This ratio not only varies

between the experiments, but also between different areas of the laser exposure. We succeeded in producing the desired ETs of different sizes only 3 times out of about 20 attempts, in each case the transformed spot was scanned in multiple positions.

Supplementary note 2. Raw STM images and ET dimensions.

Here we show the full images from which the triangles in the main text are taken. Among the large number of observed triangles, we have specifically chosen a few to show in the main text, paying attention to show the most representative examples. The triangles in the main paper thus cover various sizes of the triangles, which show different ordering patterns. In the main text we only discuss the perfect equilateral triangles, even though the 1T/1H areas that are created using the laser pulse cover many more possible shapes, which are discussed in the Supplementary Note 3.

The dimensions of the triangles are presented in Table 1 in terms of nanometers and the number of unit cells (l is given both in terms of 1T and 2H polytype unit cell dimensions for comparison.).

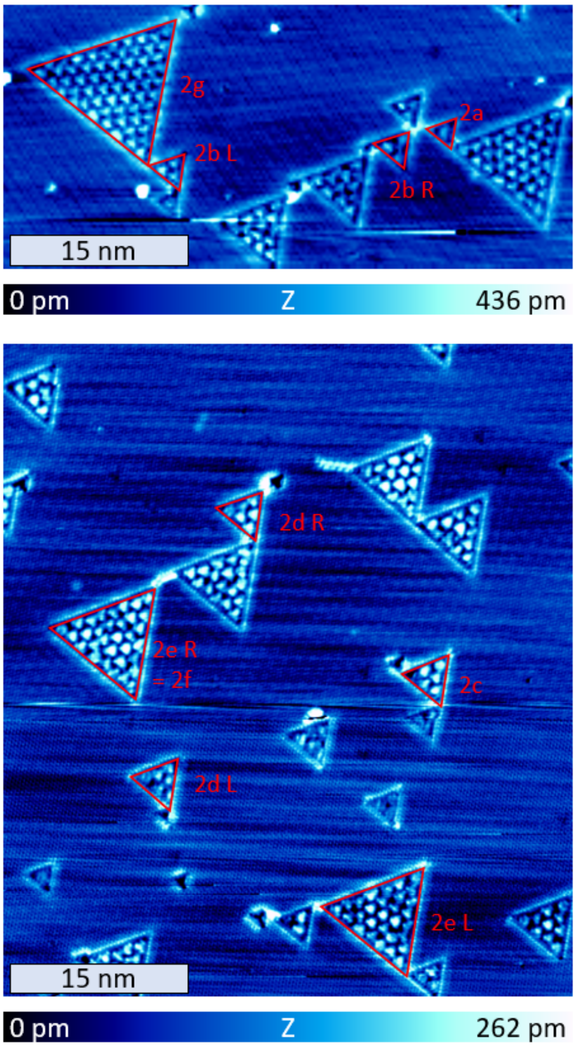

Supplementary Figure 2: Raw STM data. STM scans of the small triangular 1T areas embedded within a large 1H area. Red triangles mark the triangles that are presented in the main text.

	l (nm)	No. of 1T unit cells	No. of 2H unit cells
2a	2.69	8.02	8.09
2b R	3.36	9.99	10.08
2b L	3.36	9.99	10.08
2c	4.32	12.86	12.98
2d R	4.37	13.01	13.13
2d L	4.37	13.01	13.13
2e R	9.46	28.15	28.40
2e L	9.36	27.85	28.10
2f	13.02	38.74	39.09

Supplementary Table 1. ET size measurements l of the triangles presented in Figure 2 of the main text.

Supplementary note 3. Areas of different shapes and larger 1T areas.

Both 1T and 1H polytypes have a triangular lattice symmetry and it is only natural that the borders between the polytypes within a single layer will take on 60 and 120 degree angles. This, however does not limit the structures only to triangles, which are extensively discussed in the main text, but allows also for countless possibilities of zig-zag borders and shapes that are seemingly composed of many triangles. The 1T regions can be very small (triangles of only a few atoms on the side), to very large, covering most of the surface with only an occasional 1H triangle. In the very large 1T regions, where the borders do not anymore have a significant effect, we see that the electron ordering mostly resembles that of the top surface in a bulk material (C state or photoinduced H state). We have focused our modelling only on the perfect equilateral triangles for two main reasons. Firstly, the more complex shapes are usually larger and the borders do not have a significant effect and are thus less interesting for a theoretical analysis. Secondly, the borders of arbitrary shapes are increasingly difficult to model with each additional considered shape, while the triangles represent a clean toy model.

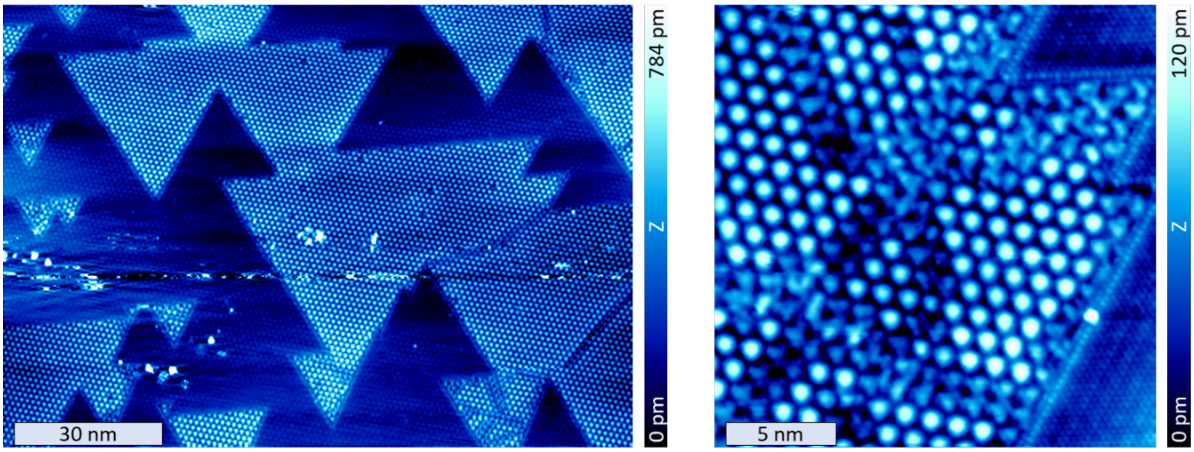

Supplementary Figure 3. STM scans of the large 1T areas intermixed with large 1H areas. Left image shows the 1T polytype in the commensurate state at 80 K, while the right image shows it in the photoinduced hidden state at 4K. In both cases we can see the disordered polarons appearing especially in the corners.

Supplementary note 4. STM measurements below 75K.

The bulk 2H-TaS_2 has a CDW transition temperature at 75 K[3], but the transition temperature to the CDW state in the single layer 1*H* phase is not completely clear. In a previous STM tip switching report, the CDW in single transformed 1*H* layer was not seen at temperatures as low as 6.5 K[4]. This could either be due to the poor tip resolution or inappropriate scanning conditions (the 3 × 3 CDW is weak and the authors have likely optimized the tip conditions to have the best signal from the investigated 1T polytype, rather than from 1H, which in their case was just a byproduct). Another very likely possibility is that the 3 × 3 CDW does not appear in the small confined areas. This is however not discussed in their paper, as the transformed part was only found as a byproduct of electronic switching to the hidden state. We have performed the top layer 1T->1H transformation also at 4 K, where we were able to observe the 3 × 3 CDW modulation in the top 1H layer, however it was much weaker than the modulation in the 1T polytype and not easily observed everywhere. In either case the appearance (or absence) of the CDW in the 1H layer does not seem to influence the electronic ordering within the triangles.

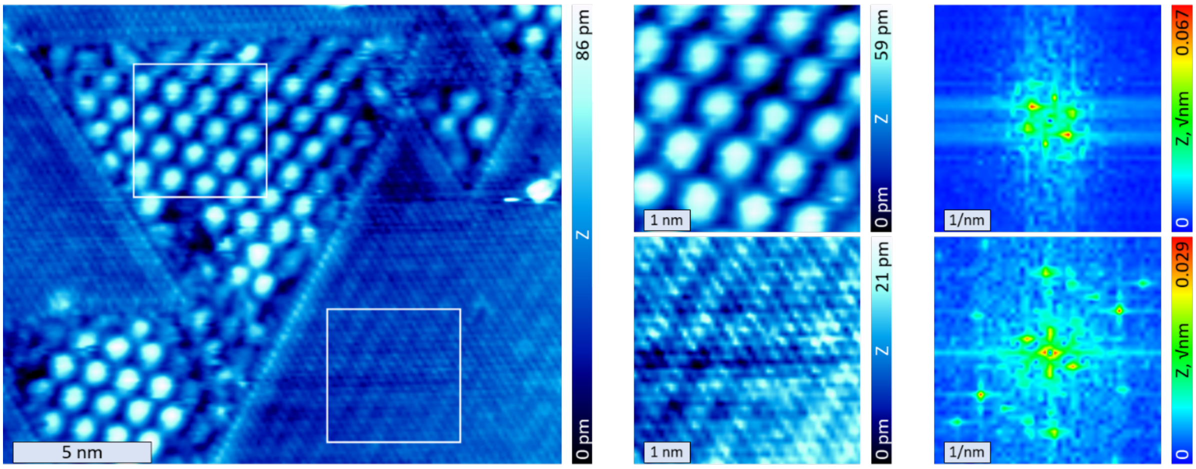

Supplementary Figure 4. Mixture of 1T and 1H polytypes at 4 K. (left) We can see both the $\sqrt{\mathbf{13}} \times \sqrt{\mathbf{13}}$ CDW in the 1T polytype and the $\mathbf{3} \times \mathbf{3}$ CDW in the 1H polytype. The images in the middle show the zoomed in region of the 1T (top) and 1H (bottom) polytype. The images on the left show their respective FFTs.

Supplementary note 5. STM imaging at different bias voltages

With changing the bias voltage for STM imaging, we can make different features in the material better visible. Here we show a few different cases of STM imaging on the border between the 1T and 1H polytype. In the 1H regions (bottom right in all three images in Supplementary Figure 5 and top left in all 4 images in Supplementary Figure 6) we see that the CDW in the C state from the bottom 1T layers is in general the best visible at slightly positive voltages. More precisely, we see that the commensurate CDW from the bottom 1T layers is best seen at +0.1 V. In the 1T region (top left in all three images in Supplementary Figure 5 and bottom right in all 4 images in Supplementary Figure 6), the best way to observe individual polarons is to scan at voltages below -0.5 V. For the best contrast when scanning the 1T polytype, we use the bias voltage of -0.8 V (most of the figures in this paper were indeed taken at -0.8 V). When changing the voltage towards zero, the relative contribution of the bands in the polarons gets lower and certain features become more pronounced. This shows as the shapes of polarons slightly change and sometimes other modulations become visible. In Supplementary Figure 5. we can see that some polarons become brighter and that there are additional states between the polarons that get much better visible at voltages close to zero. In Supplementary Figure 6, we see that only certain regions of the 1T polytype become highlighted at very low voltages.

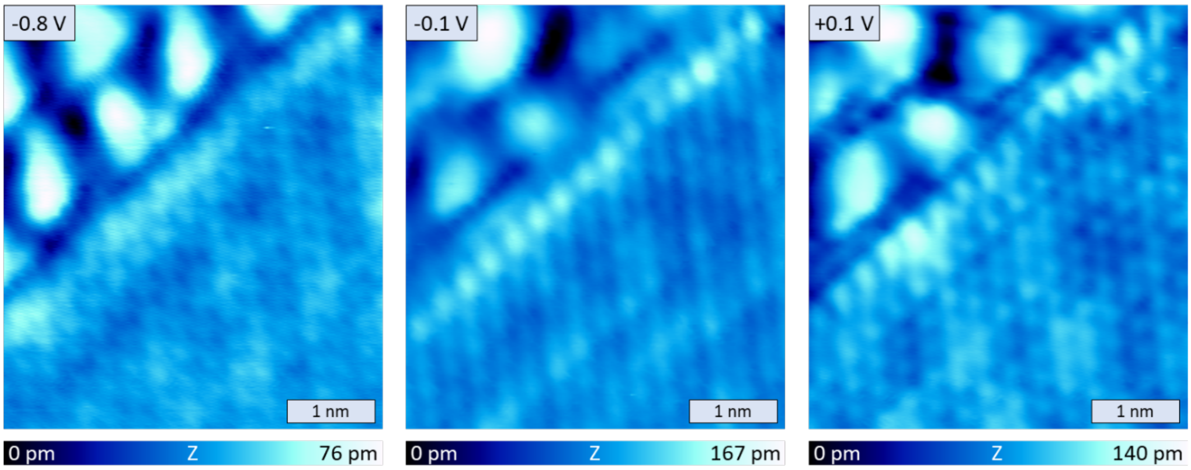

Supplementary Figure 5. Constant current STM scans of the border between the 1*T* and 1*H* polytypes at different voltages. We can see that in the 1T polytype, different polarons are brighter at different voltages. In 1H polytype, we can see that the CDW from the bottom 1T layer is best visible at +0.1V.

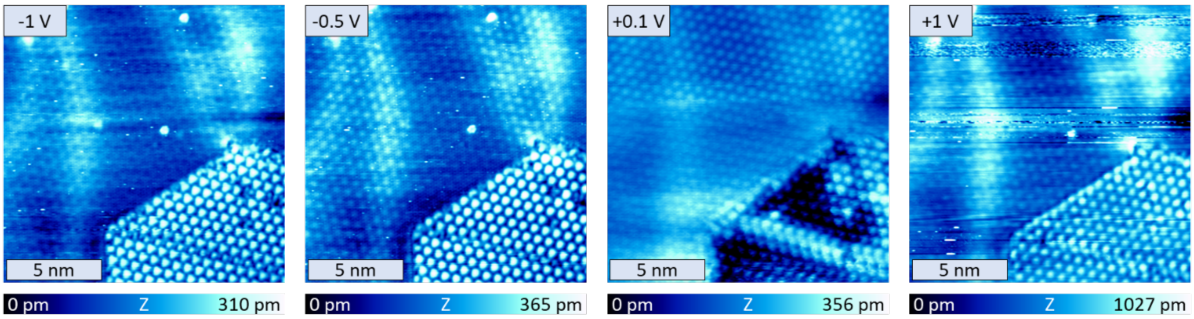

Supplementary Figure 6. Border between 1H and 1T polytype. In this case the surface was fully transformed to 1H polytype and only a small 1T part was later reversely transformed by the STM tip[2]. The stripes on the 1H polytype appear in very large transformed regions due to the mismatch between the lattice unit cells. Same as in Supplementary Figure 5, we can see that the bottom layer CDW is the best seen through the 1H layer at 0.1 V. In the 1T region, we can see that slightly positive voltages make domain walls stand out.

Supplementary note 6. Interlayer stacking

At certain bias voltages (typically within 0.1 V around zero or slightly above that) we can very clearly see the CDW modulation from the untransformed 1T polytype bottom layers, (Supplementary Figure 5 and 6). Due to the fact that we can "see through" one single 1H layer, we directly uncover the interlayer stacking of the CDW in the z-direction, by comparing the positions of the polarons in the bottom layer with the positions of polarons in the top layer. The stacking is found to be both indirect (with the polarons not directly above each other in the neighboring layers, shown in Supplementary Figure 7a) and direct (with the polarons directly above each other, shown in Supplementary Figure 7b). We attribute the stacking to the parameters such as pinning impurities, which depend on the sample growth conditions and we cannot directly and controllably repeat. It is worth mentioning that when transforming one (or more) of the layers to the textured hidden state, the stacking varies if the domain walls are not directly above each other, which was found to be the case. We clearly see this from Figure 5c in the paper by Ma et. al[4], where they induced a single triangle within a larger area of the metallic textured hidden state of the 1T polytype. In the image, we see the top two layers of the hidden state with variable vertical stacking of polarons. Furthermore, when we observe various domains of different chirality, this necessarily leads to the misalignment of the polarons between the layers, except for the special case, where the domain in the bottom layer is exactly the same as the one in the top layer, which is extremely unlikely.

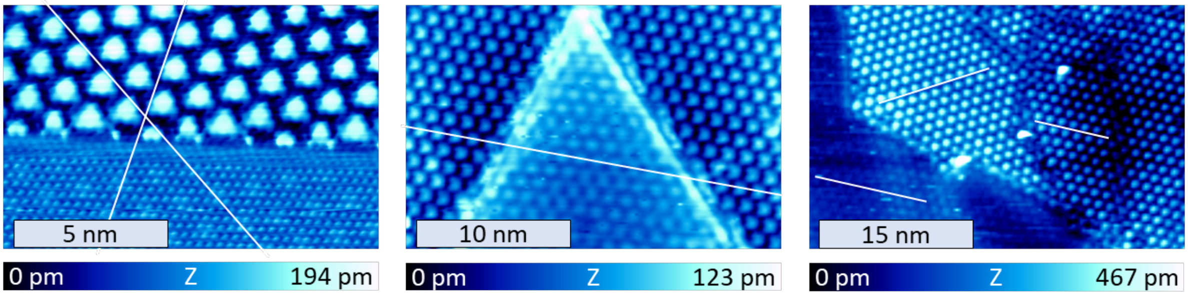

Supplementary Figure 7. The polaronic modulation of the bottom 1*T* C phase. a) The polarons are not vertically above each other and b) the polarons are vertically stacked above each other. In c) we show domains of different chirality in the top layer, while the bottom layer likely has only one ordering. The white lines are aligned with the polarons and serve as a guide to the eye.

Supplementary note 7. Band structure of the 1T/1H monolayer junction.

The band alignment diagram of the 1T/1H junction at T=77 K, assuming on spatially homogeneous metal-semiconductor junction, is shown in Supplementary Figure 8a. (For the sake of argument, we assume that the initial band structure of 1H-TaS_2 is the same as 2H-TaS_2.) At this temperature, the 1H-TaS_2 is above its ordering temperature, and is assumed to be metallic like its parent 2H compound, while the 1T phase supports a charge density wave (since $T_{CCDW\downarrow} = 140\ K$). Accordingly, the STM image (Supplementary Figure 8b) shows a uniform density on the 1H side, and a characteristic $\sqrt{13} \times \sqrt{13}$ charge modulation on the 1T side.

The edge state. The work function ϕ for 1T-TaS_2 is larger than for 2H-TaS_2, $\phi_{2H-TaS_2} < \phi_{1T-TaS_2}$, so electron injection from the 1H-TaS_2 metal into the 1T semiconductor can take place, and the junction is expected to be ohmic. Due to the fact that $\phi_{1T-TaS2} - \phi_{2H-TaS_2} < \Delta/2$, where Δ is the gap in 1T-TaS_2, upon the alignment of the Fermi levels, the charge carriers in the conduction band of the 1T-TaS_2 form a 'pocket' of width w (orange), which implies the formation of a charged edge state along the boundary. The width of the resulting metallic wire w is related to the screening length. Note that the edge state is susceptible to self-organization of charge. On a phenomenological level, the boundary presents an effective confining potential barrier $\Phi_B \simeq \Delta/2$ for electrons on the 1T-TaS_2 side.

The STM image shown in Supplementary Figure 8b clearly reveals the presence of the edge state along the 1T/1H boundary, with a width $w = 0.5{\sim}1$ nm, consistent with previous observations by STM at the boundary of the gapped C phase and the metallic phase of 1T-TaS_2 (see for example paper by Ma et al.[4]). The charge density wave order in the 1T state appears to be unperturbed beyond ~ 1.5 nm from the interface. At the edge, on the 1T side, the $\sqrt{13} \times \sqrt{13}$ commensurate CDW pattern appears distorted by the presence of the boundary, which is at an angle of 13.5 degrees with respect to the CCDW charge ordering vector.

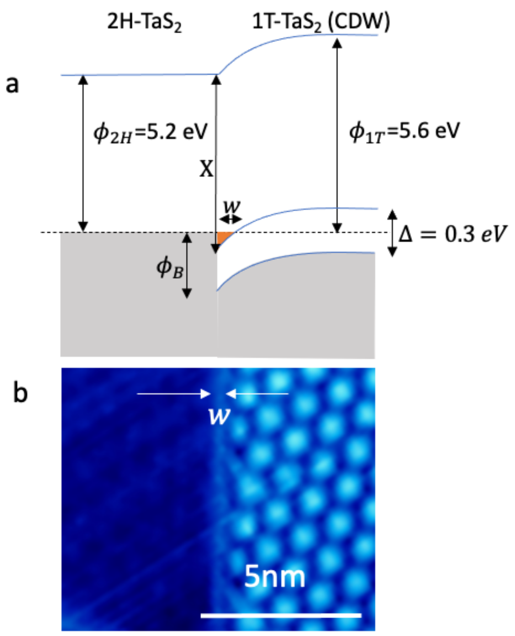

Supplementary Figure 8. The in-plane line boundary between semiconducting 1T-TaS$_2$ and metallic 1H-TaS$_2$ at 77 K. a) Schematic diagram of a metal-semiconductor ohmic contact. b) An STM image showing the edge state of width $\boldsymbol{w}$ on the boundary of the 1T and 1H monolayers.

Supplementary note 8. A quantum billiard with a CDW potential (QB+V model): model prediction of the observed salient features.

The QB calculation entails the solving of the Schrödinger equation $-\frac{\hbar^2}{2m}[\Delta_{x,y} + V(x,y)]\psi(x,y) = E\psi(x,y)$ with Dirichlet boundary conditions in the shape of an ET either for free particles (noninteracting single-electron billiards, V=0), or with an underlying 2D potential $V(\boldsymbol{r})$, imposed by interaction with the CDW in the layer below of the form: $\rho(\boldsymbol{r}) = e^{i\boldsymbol{q}_1.\boldsymbol{r}} + e^{i\boldsymbol{q}_2.\boldsymbol{r}} + e^{i\boldsymbol{q}_1.\boldsymbol{r}}$, where $\boldsymbol{q}_i$ are the CDW wavevectors, at an angle of $\pi/3$ to each other. The CDW is rotated with respect to the crystal lattice (and edges of the ET) by $\phi = 13\ °$, corresponding to the $\sqrt{13} \times \sqrt{13}$ CCDW, where $\frac{2\pi}{q_1} = 3\boldsymbol{a} + \boldsymbol{b}$, as shown in Figure 1 of the main text. Thus $V(x,y) = -V_0 Re[\rho(\boldsymbol{r}')]$, where $\boldsymbol{r}' = \begin{pmatrix} \cos\phi & -\sin\phi \\ \sin\phi & cos\phi \end{pmatrix}\begin{pmatrix} x \\ y \end{pmatrix}$, to take into account the rotation of the CDW.

The solutions of the QB+V model are shown in Figure 4 of the main paper and in the Supplementary Figures 8 and 9.

The dependence on the potential. The dependence on $|V_0|$ in units of t ($\frac{\hbar^2}{2m} = 1$) is shown in Supplementary Figure 9, ranging from $|V_0| = 0$ to 5t. Note the appearance of a 13 degree twist of the QI pattern at $|V_0| = t$ which becomes more pronounced with increasing V_0.

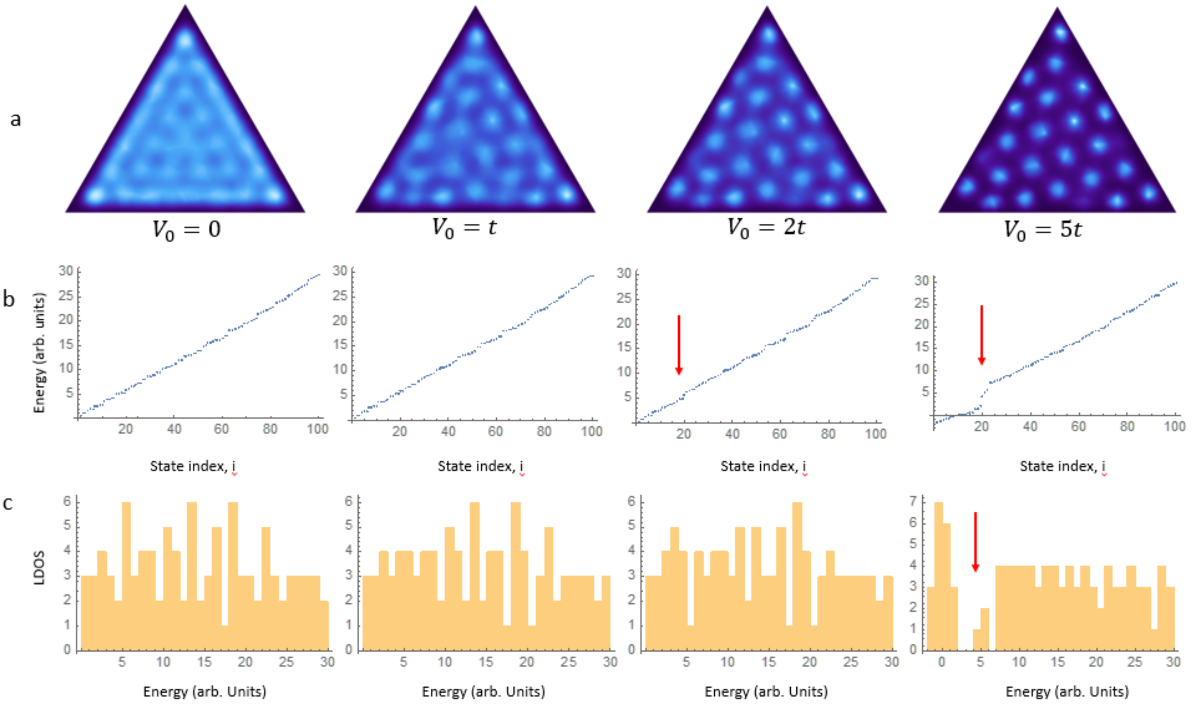

Supplementary Figure 9. Model calculations of spatial probability density, energy as a function of state index and LDOS spectrum showing a gap. A calculation of the QB+V model for different values of $|\boldsymbol{V_0}| = \boldsymbol{0} \dots \boldsymbol{5t}$. a) a plot of the eigenfunctions $\boldsymbol{\Sigma_i} |\boldsymbol{\psi_i}|^2$ within the ET where $\boldsymbol{N} = \boldsymbol{1} \dots \boldsymbol{32}$, and $\boldsymbol{N} = \boldsymbol{32}$. The twist in the pattern is clearly visible with increasing $\boldsymbol{V_0}$. b) The eigenstate energy $\boldsymbol{E_i}$ as a function of $\boldsymbol{i}$ for different values of $|\boldsymbol{V_0}|$. The gap for t/V>0.5 is indicated by the arrow. c) the LDOS as a function of energy $\boldsymbol{E}$ for different values of $|\boldsymbol{V_0}|$.

In Supplementary Figure 9b we plot the state energies E_i as a function of state index i, and in Supplementary Figure 9c we plot the local density of states (LDOS), presented with states binned together in the form of a histogram. With increasing V_0, as expected, the periodic potential creates a gap in the spectrum (shown by the arrow). It is perhaps surprising that a rotation of the QPI pattern is seen already for $V_0 = t$, while a clear gap in the LDOS appears only for much larger values of V_0 =5 (arrow). A jump in the state energy as a function of i is visible already at $2t$, but there are other jumps at higher energies which are hard to distinguish from the fundamental gap.

QI pattern of states below the gap. In Supplementary Figure 10 we plot the LDOS spatial pattern $\Sigma_{i=1 \dots N} |\psi_i|^2$ summing up to different values of N to show the QI pattern for states that appear below and above the external CDW-induced gap. The LDOS is plotted in the insert, indicating the centre of the gap at state index $i = 5$. Thus summing up the eigenstates up to $N = 5$, we can plot the states below the gap that contribute to the QI pattern. Clearly the dominant pattern is already visible, but some spots are missing around the edges, which clearly have higher energy. Understandably, higher energy (shorter wavelength) states are

necessary to fill in the space around the boundary. For $N = 32$, the LDOS spatial pattern fills all available space. With $N = 49$, no additional features are observed.

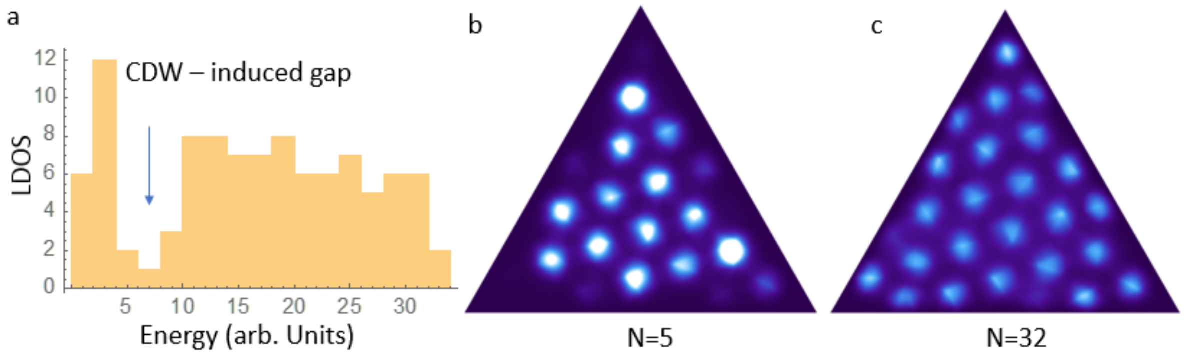

Supplementary Figure 10. **Gap in the LDOS and associated probability density plots.** A plot of the LDOS $\sim \boldsymbol{\Sigma}_{i=1...N} |\boldsymbol{\psi}_i|^2$ for different values of N, as indicated in the plots, and $V = -10$. The insert shows the LDOS as a function of energy with a clearly visible gap in the spectrum.

The QI patterns for different levels, and effective mass. The presented STM images in the main text are measured at $V = 0.8\ V$. Let us roughly estimate the relevant range of eigenvalue index for a voltage range up to 0.8 eV. Using $N = \sqrt{\frac{2m^*El^2}{\hbar^2\pi^2}}$, and assuming $m^* = m_e$, for the smallest triangle ($l = 8a = 2.64nm$), we obtain $N \approx 4$. For larger triangles, $l = 10a$, $N \approx 5$; for $l = 27a$, $N \approx 13$; and for $l = 38a, N \approx 18$. Taking into account electron-phonon coupling and polaronic effects, the effective mass may be increased. Using $m^* \simeq 4\ m_e$, this compresses the energy scale a factor of ~2. At low temperature, and in the absence of scattering and interactions of the tunneling interactions with phonons, a single eigenstate is expected to be observed in the smallest triangles (in agreement with QI pattern shown in Figure 2a of the main text).

Supplementary note 9. Confined correlated electrons in the classical limit

To simulate charges on a discrete lattice in the shape of an equilateral triangle, we assume a sceened Coulomb repulsion between charges as well as open boundary conditions at the edges of a triangle. In order to satisfy electroneutrality, the following Hamiltonian is employed (Vodeb et al, 2019, Karpov and Brazovskii, 2018):

$$\mathcal{H} = \frac{1}{2}\sum_{i,j}^{N} V'(i,j)(n_i - \bar{n})(n_j - \bar{n}), \qquad (1)$$

where $V'(i,j) = \exp(-r_{i,j}/r_s)/r_{i,j}$, r_s is the screening radius (4,5 lattice spacings) $r_{i,j} = r_i - r_j$, r_i is the i-th out of N lattice sites, n_i is the occupation number of lattice site i, the sum runs over all lattice sites and $\bar{n} = \sum_i^N n_i / N$. $\bar{n}$ represents the interaction of each charge with underlying uniformly and oppositely charged plate. Only here the plate is represented by uniformly charged lattice sites on which the charges can reside. The lattice sites form a triangular lattice in the shape of an equilateral triangle. $\mathcal{H}$ can be simplified to:

$$\mathcal{H} = \sum_{i,j}^{N} V(i,j) n_i n_j, \qquad (2)$$

where $V(i,j) = \frac{1}{2}V'(i,j) - \frac{1}{2N}\sum_k^N\big(V'(i,k) + V'(k,j)\big) + \frac{1}{2N^2}\sum_{k,l}^N V'(k,l)$. With this simplified version we simulated a fixed number of charges on differently sized triangles. The classical Monte Carlo method used has been described previously[5]. Since we were interested in what happens in the vicinity of the $f = number\ of\ charges/N = 1/13$ state, we initialized all the simulations in the $1/13$ state. The main features predicted by the model are shown in the main text.

1. Wang, Z. *et al.* Surface-Limited Superconducting Phase Transition on 1T-TaS2. *Acs Nano* **12**, 12619–12628 (2018).

2. Ravnik, J., Vaskivskyi, I. & Gerasimenko, Y. Strain-Induced Metastable Topological Networks in Laser-Fabricated TaS2 Polytype Heterostructures for Nanoscale Devices. *ACS Applied Nano* **2**, 3743–3751 (2019).

3. Hall, J., Ehlen, N., Berges, J., Loon, E. van & ACS, C. van E. Environmental Control of Charge Density Wave Order in Monolayer 2H-TaS2. *ACS Applied Nano* **13**, 10210–10220 (2019).

4. Ma, L. *et al.* A metallic mosaic phase and the origin of Mott-insulating state in 1T-TaS2. *Nature Communications* **7**, 1–8 (2016).

5. Vodeb, J. *et al.* Configurational electronic states in layered transition metal dichalcogenides. *New J Phys* **21**, 083001 (2019).
